# Exploring the role of iron-solubilizing *Bacillus* sp. in promoting cereal growth

**DOI:** 10.3389/fmicb.2025.1699358

**Published:** 2025-11-24

**Authors:** Azhar Hussain, Maryam Saeed, Hammad Anwar, Abubakar Dar, Muhammad Imran, Hossam S. El-Beltagi, Usman Zulfiqar, Obidjon Abdulloev, Nazih Y. Rebouh, P. V. Vara Prasad

**Affiliations:** 1 Department of Soil Science, The Islamia University of Bahawalpur, Bahawalpur, Pakistan; 2 Soil & Environmental Sciences Division, Nuclear Institute for Agriculture & Biology, Faisalabad, Pakistan; 3 Department of Agricultural Biotechnology, College of Agriculture and Food Sciences, King Faisal University, Al-Ahsa, Saudi Arabia; 4 Department of Agronomy, Faculty of Agriculture and Environment, The Islamia University of Bahawalpur, Bahawalpur, Pakistan; 5 Department of Chemistry, Andijan State University, Andijan, Uzbekistan; 6 Institute of Environmental Engineering, RUDN University, Moscow, Russia; 7 Department of Agronomy, Kansas State University, Manhattan, KS, United States

**Keywords:** *Bacillus*, biofortification, maize, micronutrient, wheat, iron

## Abstract

**Introduction:**

Iron (Fe), being the most limited micronutrient in soils, performs key functions in a plant’s physiology, namely, enzyme activation and chlorophyll synthesis. Its deficiency prevails in humans in the form of disorders in pregnant women and children, for example, anemia.

**Methods:**

Therefore, the current investigation aims at isolating, screening, characterizing, and identifying Fe-solubilizing bacteria and their impact on maize and wheat growth under axenic conditions.

**Results:**

The results depicted their differential response against siderophores and exopolysaccharide production, urease activity, phosphorus, and zinc solubilization. Under axenic conditions, the maximum increase in wheat shoot, root length, chlorophyll a, chlorophyll b, and carotenoid contents under AH-22 isolates was observed, which showed an increase of 67.2, 34.6, 24.7, 30.1, and 41.7%, respectively, compared to the control. Similarly, maximum increase of 41.8, 41.7, 37.2, 37, and 16.4%, respectively, was recorded in maize shoot and root lengths, chlorophyll a, chlorophyll b, and carotenoid contents under AH-34 strain inoculated treatment. Furthermore, the molecular identification of the promising rhizobacteria revealed that AH-22 was identified as *Bacillus subtilis*, AH-26, AH-36, AH-46 as *Bacillus* sp., and AH-34 as a *Bacillus megaterium* strain.

**Discussion:**

On the basis of the revealed results, it can be concluded that rhizobacterial strains *B. subtilis* (AH-22) in wheat and *B. megaterium* (AH-34) in maize effectively enhanced wheat and maize growth by improving nutrient solubilization and physiological traits. Moreover, the studied strains need to be tested in natural field conditions, and the development of certain formulations to boost growth and Fe-biofortification in cereals.

## 1 Introduction

In the food chain, iron (Fe) plays a significant role as a micronutrient. Besides this, Fe is required in very small amounts; however, even a small amount of usage in dietary food can create very serious health problems (Ul Hussan et al., 2022). Deficiency of Fe is a serious health issue, and globally, around two billion people are affected by it, particularly in developing nations (Vasconcelos et al., 2017). Particularly in pregnant women and children, Fe deficiency can cause serious problems like anemia, which ultimately adversely impacts cognitive growth and weakens or slows down the immune system (Lopez et al., 2016). Riaz et al. (2020) reported that Fe, as an essential part of chlorophyll pigment, performs a significant role in plant’s biochemical and physiological pathways and enhances livestock and human health. With the rise of the global population, adverse impacts of climate change, environmental stress, and soil calcareousness highlighted Fe deficiency as an emerging challenge for sustainable agricultural production and food security (Tanin et al., 2022). Zulfiqar et al. (2020) described that the deficiency of Fe in Pakistani soils is prevalent, and approximately 30% of the soil is calcareous due to its high pH and presence of bicarbonates.

Therefore, it is the dire need of the hour to address Fe deficiency sustainably to cope with Fe malnutrition in humans (Xie et al., 2020). Biofortifying cereal crops with Fe presents a promising, sustainable, and long-term solution for increasing human health and mitigating nutritional security. Dietary diversification, Fe fortification, and supplementation are important techniques to reduce Fe deficiency problems (da Silva Lopes et al., 2021). Scientists described two plant categories, that is, gramineous and non-gramineous, the latter adopts reduction as Fe-solubilization through the extrusion of a proton by roots and phenolic compounds for conversion of Fe^3+^ (ferric ion) to Fe^2+^ (ferrous ion) and enhances availability for plants (Nathiya et al., 2020). Moreover, another option is the adoption of selective breeding to develop wheat and maize cultivars with high biofortification potential, thereby enhancing food quality and addressing malnutrition in children and adults (Sheoran et al., 2022).

Wheat and maize are important cereal crops in Southeast Asia, especially Pakistan, and play a critical role in global food security. These are the two most widely cultivated and consumed cereal grains worldwide (Ahsin et al., 2020). Wheat, a staple in many regions, is a primary ingredient in bread, pasta, and various baked goods, providing essential carbohydrates, proteins, and nutrients. Its adaptability to diverse climates makes it a reliable food source across continents (Kaushal et al., 2023). Maize, also known as corn, is equally significant, serving as a direct food source, a key ingredient in processed foods, and a major component of animal feed. This versatility supports both human nutrition and livestock production, ensuring a steady food supply (Sarwar and Biswas, 2021). Together, wheat and maize significantly contribute to global caloric intake and nutrition, making them indispensable in combating hunger and ensuring food stability, particularly in developing nations (Oluwole et al., 2023).

In cereal crops, the use of microbes that produce siderophores for efficient Fe uptake and effective Fe translocation should be integrated (Anwar et al., 2022). The ability of plant growth-promoting rhizobacteria (PGPR) to produce siderophores facilitates enhanced Fe uptake in cereals. The secretions of different organic acids are also involved in Fe-solubilization by lowering microsite pH in alkaline calcareous soils (Mushtaq et al., 2024). Further, Khalid et al. (2015) described that siderophores’ functions comprise an improvement in soil fertility, plant health, and crop growth. In addition, different strains of PGPR inoculation with crop plants not only improve various medicinal plant qualities like spice crops (Sahay and Patra, 2014) but also increase cereal yield (Kumar et al., 2013). Zhang et al. (2022) described that mobilization and translocation of Fe in plants commonly occur through (i) the chelation process of Fe with Phyto-siderophores and (ii) in the form (Fe^+2^) free ions of iron. Rhizobacteria affect the growth of plants either directly or indirectly in the rhizosphere or by root colonization (Hassanisaadi et al., 2021). Mushtaq et al. (2021a) also confirm their role in the rhizosphere in improving nutrient use efficiency, plant growth, physiological processes, and yield (Mehmood et al., 2018) by fixing atmospheric nitrogen (El-Sayed et al., 2014), solubilization of phosphorus, extracellular enzymes synthesis, namely, catalase, oxidase, lipase, chitinase, and protease, phytohormones secretions, namely, auxins, gibberellins, etc., and exopolysaccharides production (Mumtaz et al., 2017). Moreover, El-Sayed et al. (2014) also mentioned growth promotion by indirectly controlling fungal pathogens, namely, *Sclerotinia sclerotium*, *Fusarium oxysporum*, and some species of nematodes such as *Meloidogyne incognita*, by the indirect mechanism of quenching Fe (a potent micronutrient for pathogens) through siderophores production.

The previous investigations primarily focus on the single mechanism of Fe-solubilization (siderophore production) by bacteria and enhancing growth, Fe uptake, and its biofortification, that is, Ahmed and Holmström (2014) and Schalk (2025). Similarly, Dimkpa et al. (2009) and Sarwar et al. (2022) also demonstrated the siderophore-mediated Fe-solubilization by *Pseudomonas*, *Azotobacter,* and Bacillus subtilis species. Therefore, the novelty of the present investigation lies in the bacterial multifarious approach by not only solubilizing Fe but also phosphorus (P), zinc (Zn), and exopolysaccharides (EPS) production, and urease activity by broadening the scope of nutrients mobilization in cereals’ rhizosphere than previous reports.

Thus, the present investigation aims to isolate and screen siderophore-producing bacterial isolates and assess their potential for improving the growth and physiology of maize and wheat crops under controlled conditions. After that, these isolates were identified genetically (16S ribosomal RNA [rRNA] gene sequencing), and then these strains were characterized for plant growth-promoting (PGP) and biochemical characteristics. So, it can be hypothesized that the Fe-solubilizing bacteria can improve the growth and physiological attributes of maize and wheat.

## 2 Materials and methods

### 2.1 Isolation of Fe-solubilizing bacterial strains from the rhizosphere of maize and wheat

Soil samples from the maize and wheat rhizosphere were collected in clean bags and stored at 4 °C to isolate bacteria using the serial dilution technique, a standard method described by Wollum (1982). The serial dilutions were spread on nutrient agar medium and incubated for 24 h at 30 ± 2 °C. Thirty fast-growing isolates, each from maize and wheat, were purified and stored at −20 °C in 50% glycerol stock.

### 2.2 Screening of bacteria by Fe-solubilization and growth-improving traits

First, the strains were screened based on siderophore (Fe-loving compounds) production by isolated bacterial strains, which was determined through spot inoculating on chrome-azurol S (CAS) media amended with FeO and iron ash (insoluble Fe sources) and incubating at 30 ± 2°sC for 48 h (Schwyn and Neilands, 1987). The appearance of orange halo zones is considered as siderophore production. Second, the screening was conducted based on plant growth-promoting (PGP) traits, such as urease activity, exopolysaccharides production, and P and Zn solubilization. Similarly, the strains were spot-inoculated on Pikovskaya’s agar medium and incubated for 72 h at 30 ± 2 °C for the appearance of clear halo zones around microbial colonies as described by Pikovskaya (1948). Christensen’s urea broth medium was inoculated with the studied strains, and a color change of the medium from yellow to pink is an indication of urease activity in strains, as mentioned by Cappuccino and Sherman (2002). While for the EPS production, bacterial strains were spot-inoculated on Reference Change Value (RCV) glucose agar and incubated for 48 h at 30 ± 1 °C to note the mucoid growth around the colonies (Strieth et al., 2021). Moreover, zinc solubilization was confirmed by the appearance of clearing zones around bacterial colonies after 72 h of spot inoculation on tris-minimal salt media amended with 0.1% zinc oxide (Fasim et al., 2002). Fifteen isolates from both crops were selected on the basis of PGP traits for evaluation in the axenic conditions jar trial.

### 2.3 Impact of Fe-solubilizing rhizobacteria on the growth of wheat and maize in axenic conditions jar trials

Maize and wheat trials were conducted in the growth room of the Department of Soil Science, The Islamia University of Bahawalpur. The seeds of maize (Pioneer P1543 cultivar) and wheat (Ghazi-19 cultivar) were purchased from the local grain market of Bahawalpur, Pakistan. The seeds of wheat and maize were surface disinfected by dipping in HgCl_2_ (0.2%) for 3 min and 95% ethanol for 1 min, followed by 6 times washing with sterilized distilled water as described by Al-Adham et al. (2013). The culture of 15 rhizobacterial strains, each from wheat and maize, was prepared in Luria–Bertani (LB) broth medium and adjusted to an optical density of OD_600_ = 0.5 (10^8^ colony-forming units [cfu] mL^−1^).

Seed inoculation was performed by immersing the surface-disinfected seed in the culture of the respective Fe-solubilizing isolate for 30 min before sowing, while the control seeds were dipped in sterilized broth. Plastic jars (20 cm height, 10 cm width/diameter) were filled with 600 g of sand and soaked with half-strength Hoagland solution to maintain field capacity and autoclaved twice for sterilization. After cooling, the seeds were sown in jars (6 seeds of maize and 10 seeds of wheat) in a completely randomized design (CRD) with 3 replications. The conditions in the growth room were set as 12 h dark at 15 ± 2 °C and 12 h light at 25 ± 2 °C temperature, with 70–90% humidity, and for maize and 14–28 °C with 60–75% humidity for wheat. A half-strength Hoagland solution was used to irrigate the jars to meet the nutritional requirements.

After 25 days of germination, shoot length (cm), root length (cm), and dry weight (g) of root and shoots were determined. Chlorophyll a, chlorophyll b, and carotenoids were calorimetrically determined by taking readings on a spectrophotometer at 660, 665, and 470 nm, respectively, while their concentrations were measured using the formulae provided by Arnon (1949) and Wellburn (1994). The root colonization of rhizobacterial strains was performed using the method described by Simons et al. (1996). The 1 cm of the root surface was cut with a sterile blade and dipped in sterile distilled water, and the serial dilution method was used to count the microbial colonization in cfu cm^−1^ of root surface (Dar et al., 2022).

### 2.4 Determination of Fe-solubilization index and efficiency

The five prominent strains (both from wheat and maize) were selected based on growth-promoting studies and subjected to screening against the Fe-solubilization index (Fe-SI) and efficiencies (Fe-SE) for biochemical and molecular characterization. The Fe-SI and Fe-SE were calculated by the equations provided by Vazquez et al. (2000) as follows:

Fe-SI=(Colony Diameter+Halo Diameter)/Colony Diameter

Fe-SE=(Halo Diameter/Colony Diameter)×100

Here, the colony and halo zone diameters were measured using a vernier caliper.

### 2.5 Identification of Fe-solubilizing isolates through 16S rRNA gene sequencing

Based on solubilization index and solubilization efficiency, five best Fe-solubilizing isolates, two from wheat (AH-22 and AH-26) and three from maize (AH-34, AH-36, and AH-46), were selected for molecular identification based on polymerase chain reaction (PCR) amplification and sequencing of partial gene 16S rRNA. Bacterial DNA was extracted using phosphate-buffered saline (PBS) buffer including proteinase-K enzyme (Mahuku, 2004), and PCR amplification of the 16S rRNA gene was performed using 27F (5′-AGAGTTTGATCMTGGCTCAG-3′) and 1492R (5′-TACGGY TACCTTGTTACGACTT-3′) universal primers. The amplified 16S rRNA PCR product has been sent to Macrogen^®^ Seoul, Korea, for partial sequencing of 16S rRNA. The phylogenetic relationship of bacterial isolates was analyzed using the Neighbor-Joining (NJ) method, as described by Saitou and Nei (1987), with MEGA X software (Kumar et al., 2024). The 16S rRNA gene sequences obtained after basic local alignment search tool-nucleotide (BLASTn) analysis^1^ were aligned with closely related reference sequences retrieved from the National Center for Biotechnology Information (NCBI) database using the MUltiple Sequence Comparison by Log-Expectation (MUSCLE) algorithm. Evolutionary distances were computed using the Maximum Composite Likelihood method (Tamura et al., 2004). The reliability of the phylogenetic tree topology was evaluated by bootstrap analysis with 1,000 replications (Felsenstein, 1985). The resulting Neighbor-Joining tree was visualized and annotated in MEGA 12. Strains showing ≥97% similarity were considered to belong to the same genus, while those with ≥99% similarity were assigned to the same species level.

### 2.6 Biochemical characterization of the selected Fe-solubilizing strains

The identified isolates were tested for different biochemical attributes, namely, oxidase and catalase activities, 1-aminocyclopropane-1-carboxylic acid (ACC)-deaminase activity, hydrogen cyanide (HCN) production ability, protease, cellulase, and indole-3-acetic acid (IAA) production. The catalase and oxidase activities of selected isolates were measured by using the standard protocol of Cappuccino and Sherman (2002). The cellulose and protease activities were evaluated using the methods discussed by Chernin et al. (1998) and Sierra (1957), respectively. Moreover, the production of indole-3-acetic acid (IAA) of Fe-solubilizing bacteria was determined both with and without L-tryptophan amendments through the colorimetric method using Salkowski’s reagent (Ehmann, 1977). ACC-deaminase activity, measured by the standard protocol of Penrose and Glick (2003), while the production of hydrogen cyanide (HCN) by selected isolates was measured by the method described by Lorck (1948).

### 2.7 Statistical analysis

For statistical analyses, the significance of the data was assessed by performing an analysis of variance (ANOVA) under the linear model of completely randomized design (CRD). By using multiple pairwise comparisons, means were compared through LSD at a 5% level of significance, using Statistix^®^ version 8.1 (Analytical Software, Tallahassee, FL, USA) following Steel et al. (1997). Moreover, the multivariate analysis, for example, principal component analysis (PCA) and Pearson’s correlation, was performed on the “Origin” software version 2025b.

## 3 Results

### 3.1 Isolation of Fe-solubilizing bacterial isolates

From the rhizosphere of wheat, 30 isolates coded as AH-1, AH-2, …, AH-30, and 30 isolates from the rhizosphere of maize coded as AH-31, AH-32, …, AH-60 were isolated and screened for Fe-solubilization qualitatively and quantitatively. Siderophore production qualitatively confirmed that 15 isolates from each wheat and maize sources have solubilized both sources of insoluble Fe (FeO and Fe ash) and were selected for growth-promoting traits (Table 1).

**Table 1:** Potential of Fe-solubilizing isolates, isolated from maize and wheat rhizosphere for siderophore production, phosphorus, urease, exopolysaccharide and zinc solubilization.

Bacterial isolates	Siderophore production with FeO	Siderophore production with Fe ash	Phosphorus solubilization	Urease activity	EPS production	Zinc solubilization
Wheat						
AH-1	+	-	+	-	+	+
AH-3	+	-	+	-	+	+
AH-4	+	-	+	+	+	+
AH-8	+	-	+	-	+	+
AH-10	+	-	+	+	+	+
AH-11*	+	+	+	+	+	+
AH-12*	+	+	+	+	+	+
AH-13*	+	+	+	+	+	+
AH-14*	+	+	+	+	+	+
AH-15*	+	+	+	+	+	+
AH-16*	+	+	+	+	+	+
AH-17*	+	+	+	+	+	+
AH-18*	+	+	+	+	+	+
AH-19*	+	+	+	+	+	+
AH-21*	+	+	+	+	+	+
AH-22*	+	+	+	+	+	+
AH-23*	+	+	+	+	+	+
AH-25*	+	+	+	+	+	+
AH-26*	+	+	+	+	+	+
AH-29*	+	+	+	+	+	+
Maize						
AH-31*	+	+	+	+	+	+
AH-32*	+	+	+	+	+	+
AH-34*	+	+	+	+	+	+
AH-36*	+	+	+	+	+	+
AH-37	+	-	+	-	+	+
AH-38*	+	+	+	+	+	+
AH-42^*^	+	+	+	+	+	+
AH-43^*^	+	+	+	+	+	+
AH-44^*^	+	+	+	+	+	+
AH-45^*^	+	+	+	+	+	+
AH-46^*^	+	+	+	-	+	+
AH-49^*^	+	+	+	+	+	+
AH-51^*^	+	+	+	+	+	+
AH-54^*^	+	+	+	+	+	+
AH-55	+	-	+	-	+	+
AH-56^*^	+	+	+	+	+	+
AH-57	+	-	+	-	+	+
AH-58	+	-	+	+	+	+
AH-59^*^	+	+	+	+	+	+
AH-60	+	-	+	+	+	+

*Bacterial strains are best in siderophore production, phosphorus solubilization, urease activity, exopolysaccharide and zinc solubilization were preferred for further study (These results are repeated in three replicates). Positive symbol (+) indicates the presence of character and negative symbol (−) indicates absence of character.

### 3.2 *In vitro* screening of Fe-solubilizing bacterial isolates

Iron-solubilizing rhizobacterial strains were further screened for urease activity, Zn solubilization, P solubilization, and production of exopolysaccharide. Results shown in Table 1 confirmed that all Fe-solubilizing bacterial isolates of wheat showed urease activity, excluding AH-1, AH-3, and AH-8, and all maize isolates showed urease activity except AH-37, AH-46, AH-55, and AH-57 (Table 1). All isolates of Fe-solubilizing bacteria were found positive for phosphorus solubilization, zinc solubilization, and exopolysaccharides production (Table 1).

### 3.3 Impact of Fe-solubilizing isolates on growth attributes of wheat

**Figure 1:**
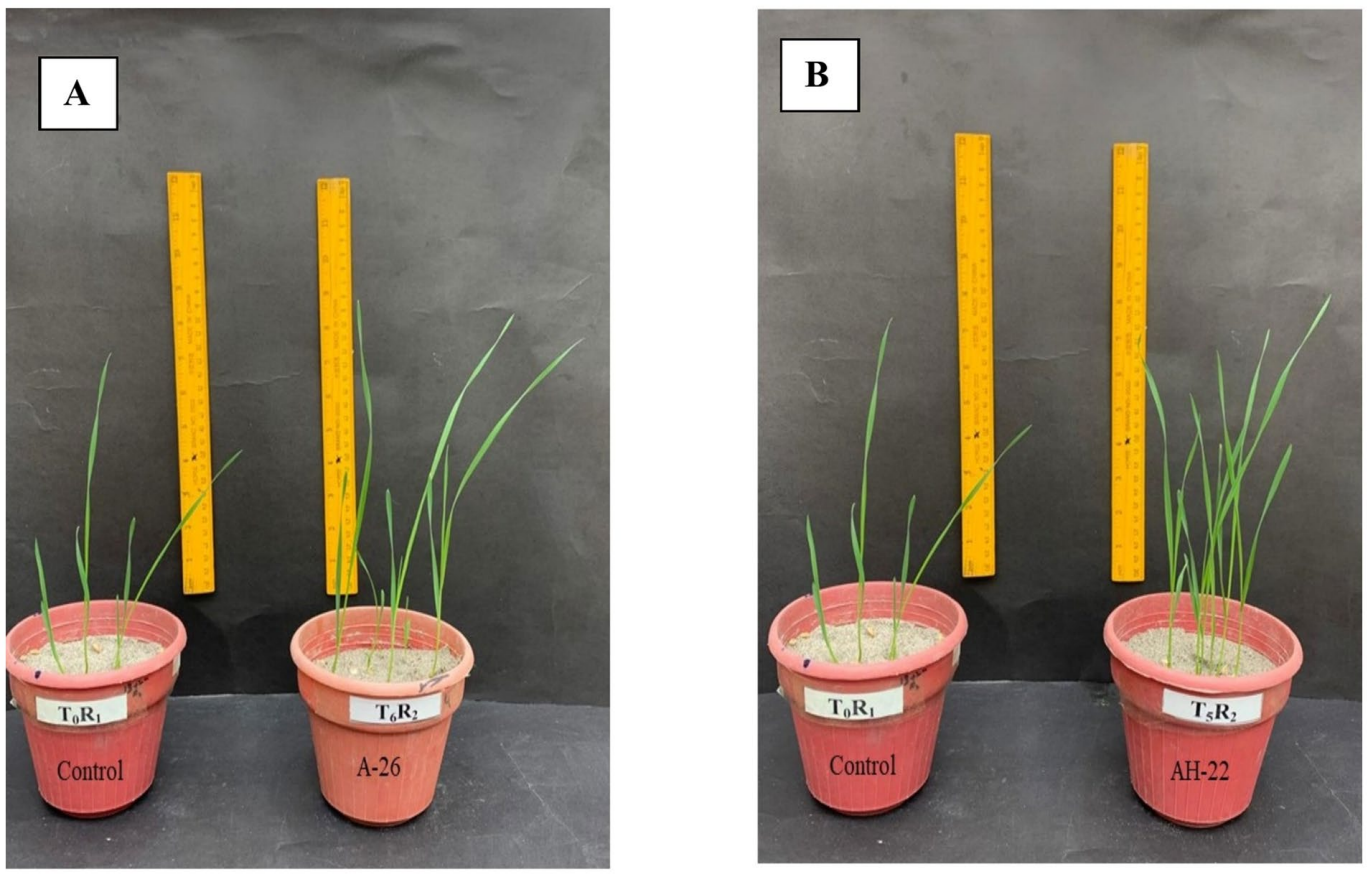
Comparison in growth of wheat by application of Fe-solubilizing bacterial isolates **(A)** AH-26 and **(B)** AH-22 compared to the control treatment.

Under control conditions, jar trial results confirm that the sole application of Fe solubilizing isolates potentially enhanced the wheat growth (Table 2 and Figure 1). Statistical analysis revealed that the highest improvement in shoot length was observed in isolate AH-22, which is 67.2% over the un-inoculated control. A subsequent improvement was observed in AH-26, AH-17, AH-19, and AH-29 isolates that enhanced the shoot length by 54.6, 44.8, 40.4, and 28.5%, respectively, over the control. The highest improvement in root length, 34.6 and 33.8%, was found under AH-22 and AH-26, followed by AH-17, AH-19, and AH-29, which showed 26.6, 21.5, and 19.3%, respectively, enhanced as compared with the control. The lowest improvement in root length was observed in the AH-18 strain, which showed a 4.4% increase over the control. Shoot dry weight was also maximum recorded in AH-22, that is 50%, followed by AH-26, AH-17, AH-19, and AH-29, which showed 47.1, 44.1, 41.2, and 44.1% improvement, respectively, as compared with the un-inoculated control. Statistical analysis confirmed that AH-26 and AH-22 showed significant increases in root dry biomass over the un-inoculated control, which showed an increase of 190 and 170%, respectively. Further improvement was observed in AH-17, AH-19, and AH-29, which showed 120, 110, and 120%, respectively, as compared with the control.

**Table 2:** Impact of Fe-solubilizing isolates inoculants on wheat growth, root colonization and chlorophyll contents under axenic conditions.

Treatments	Root length	Shoot length	Shoot dry weight	Root dry weight	Chlorophyll a	Chlorophyll b	Carotenoids	Root colonization
	(cm)		(g jar^−1^)		(μg g^−1^ FW)			cfu × 10^5^ cm^−1^
Control	5.8 ± 0.034^k^	15.3 ± 0.033^m^	0.113 ± 0.003^e^	0.033 ± 0.06^e^	0.50 ± 0.06^g^	0.52 ± 0.06^h^	0.200 ± 0.06^f^	11.0 ± 0.33^l^
AH-13	6.3 ± 0.033^g^	18.6 ± 0.023^gh^	0.127 ± 0.007^de^	0.053 ± 0.03^de^	0.52 ± 0.03^fg^	0.57 ± 0.01^d-f^	0.227 ± 0.09^c-e^	26.0 ± 0.33^i^
AH-11	6.3 ± 0.043^g^	18.5 ± 0.013^h^	0.137 ± 0.007^d^	0.063 ± 0.03^d^	0.53 ± 0.03^ef^	0.57 ± 0.03^d-f^	0.243 ± 0.03^cd^	28.0 ± 0.58^h^
AH-12	6.3 ± 0.058^g^	18.8 ± 0.016^g^	0.143 ± 0.003^b-d^	0.073 ± 0.06^ab^	0.53 ± 0.06^ef^	0.58 ± 0.03^c-f^	0.233 ± 0.03^c-e^	30.0 ± 0.5 ^g^
AH-18	6.1 ± 0.048^j^	16.5 ± 0.013^l^	0.127 ± 0.003^de^	0.047 ± 0.03^de^	0.51 ± 0.03^fg^	0.53 ± 0.09^h^	0.217 ± 0.03^ef^	14.0 ± 0.35^k^
AH-22	7.8 ± 0.033^a^	25.6 ± 0.012^a^	0.170 ± 0.006^a^	0.090 ± 0.02^a^	0.62 ± 0.02^a^	0.68 ± 0.03^a^	0.283 ± 0.03^a^	61.0 ± 0.33^a^
AH-26	7.8 ± 0.058^a^	23.7 ± 0.012^b^	0.167 ± 0.003^a^	0.097 ± 0.03^a^	0.61 ± 0.03^ab^	0.64 ± 0.06^b^	0.267 ± 0.03^ab^	59.0 ± 0.38^a^
AH-25	6.7 ± 0.046^e^	19.4 ± 0.013^f^	0.167 ± 0.003^a^	0.073 ± 0.03^ab^	0.54 ± 0.03^de^	0.58 ± 0.03^c-e^	0.247 ± 0.03^bc^	39.0 ± 0.33^e^
AH-16	6.5 ± 0.058^f^	19.2 ± 0.016^f^	0.140 ± 0.006^cd^	0.070 ± 0.03^cd^	0.52 ± 0.03^ef^	0.58 ± 0.03^d-f^	0.240 ± 0.02^cd^	36.0 ± 0.31^f^
AH-23	6.1 ± 0.058^ij^	16.8 ± 0.012^k^	0.127 ± 0.003^de^	0.043 ± 0.03^de^	0.51 ± 0.03^fg^	0.53 ± 0.03^gh^	0.227 ± 0.03^c-e^	19.0 ± 0.33^j^
AH-14	6.1 ± 0.06^h-j^	17.4 ± 0.015^j^	0.127 ± 0.003^de^	0.060 ± 0.06^de^	0.51 ± 0.06^fg^	0.56 ± 0.03^ef^	0.223 ± 0.03^de^	20.0 ± 0.35^j^
AH-17	7.4 ± 0.058^b^	22.2 ± 0.017^c^	0.163 ± 0.003^ab^	0.073 ± 0.06^ab^	0.60 ± 0.06^ab^	0.63 ± 0.06^b^	0.240 ± 0.06^cd^	49.0 ± 0.33^b^
AH-19	7.1 ± 0.033^c^	21.5 ± 0.014^d^	0.160 ± 0.001^a-c^	0.070 ± 0.09^a-c^	0.59 ± 0.09^bc^	0.60 ± 0.06^c^	0.243 ± 0.03^cd^	46.0 ± 0.31^c^
AH-21	6.2 ± 0.036^hi^	17.5 ± 0.006^ij^	0.130 ± 0.006^de^	0.060de ± 0.03^de^	0.52 ± 0.03^fg^	0.56 ± 0.06^fg^	0.227 ± 0.03^c-e^	20.0 ± 0.38^j^
AH-29	7.0 ± 0.058^d^	19.7 ± 0.028^e^	0.163 ± 0.003^ab^	0.073 ± 0.03^ab^	0.57 ± 0.03^cd^	0.59 ± 0.06^cd^	0.243 ± 0.03^cd^	41.0 ± 0.37^d^
AH-15	6.2 ± 0.058^gh^	17.7 ± 0.026^i^	0.133 ± 0.003^de^	0.060 ± 0.03^de^	0.52 ± 0.03^ef^	0.58 ± 0.03^d-f^	0.227 ± 0.03^c-e^	25.0 ± 0.33^i^
LSD (*p* ≤ 0.05)	0.09	0.26	0.02	0.01	0.02	0.02	0.02	1.95

Results shown in Table 2 revealed that application of Fe-solubilizing bacterial isolates increases root colonization under controlled conditions in wheat. Fe-solubilizing strains performed a significant improvement in root colonization over the un-inoculated control. While AH-22 showed maximum root colonization, that is, 61 × 10^5^ cfu g^−1^ root, and further increase was observed in bacterial isolates AH-26, AH-17, AH-19, and AH-29, which showed an increase of 59 × 10^5^ cfu g^−1^ root, 49 × 10^5^ cfu g^−1^ root, 46 × 10^5^ cfu g^−1^ root, and 41 × 10^5^ cfu g^−1^ root, respectively, over the un-inoculated control. In comparison, the AH-18 shows minimum improvement in root colonization, that is, 14 × 10^5^ cfu g^−1^ root. However, all the tested bacterial isolates used in this trial showed a significant improvement in root colonization over the un-inoculated control.

### 3.4 Impact of Fe-solubilizing bacterial isolates on the physiological attributes of wheat plant

Data shown in Table 2 confirmed that the use of Fe-solubilizing bacteria potentially enhanced chlorophyll a, chlorophyll b, and carotenoids under axenic conditions in wheat seedlings. The highest improvement in chlorophyll a was observed in AH-22, which showed an improvement of 24.7% and the subsequent increases were observed in AH-26, AH-17, AH-19, and AH-29, which were 22, 20, 18, and 14%, respectively. While chlorophyll b results confirm that AH-22 also shows maximum results as compared with the un-inoculated control, which showed a maximum of 30.1%, followed by AH-26, AH-17, AH-19, and AH-29, which showed a maximum of 22.4, 21.2, 16, and 12.8%, respectively. In wheat seedlings, carotenoid contents were maximum in the AH-22 isolate. Which showed a 41.7% increase over control, while further improvements were observed in AH-26, AH-17, AH-19, and AH-29, which showed an improvement of 33.3, 20, 21.7, and 21.7% respectively, over the un-inoculated control.

### 3.5 Impact of Fe solubilizing isolates on the growth of maize seedlings

**Figure 2:**
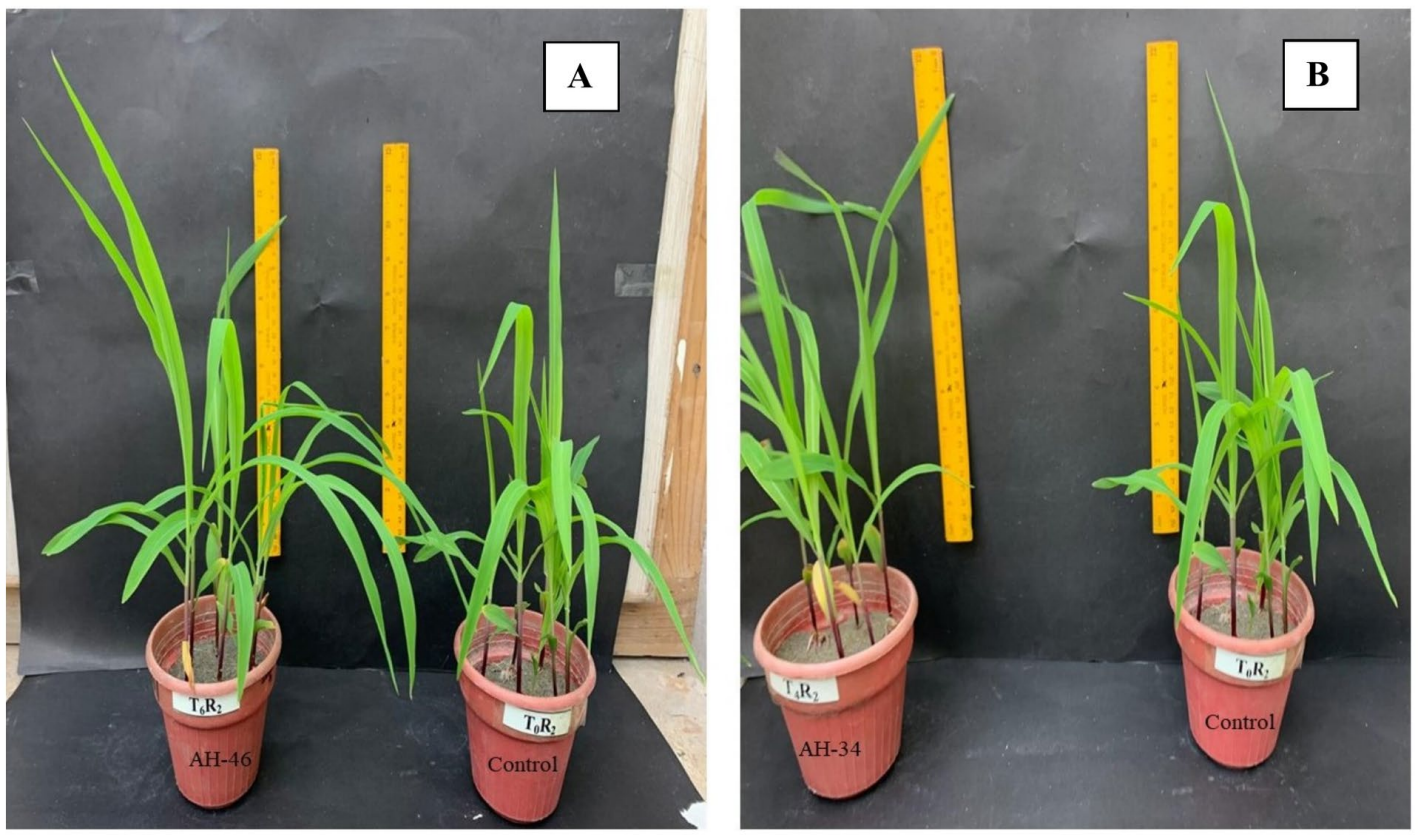
Comparison in growth of maize by application of Fe-solubilizing bacterial isolates **(A)** AH-46 and **(B)** AH-34 compared to the control treatment.

The application of Fe-solubilizing bacterial isolates to the growth attributes of maize plants under controlled conditions was significantly improved, as shown in Table 3 and Figure 2. The highest improvement in shoot length and dry weight was noted in bacterial strain AH-34, which showed an improvement of 41.8 and 71.9% respectively, over the un-inoculated control. Subsequent increase was examined in AH-46, AH-36, AH-49, and AH-44 strains, which increased the length and dry weight by 34.5 and 53.1%; 31.7 and 53.1%; 25.4 and 43.8%; 25.4 and 50%, respectively, over un-inoculated control. The highest improvement in root length, 41.8% improvement, was observed in bacterial strain AH-34, followed by AH-46, AH-36, AH-49, and AH-44, which were 41.5, 40.1, 36.7, and 33.6%, respectively, over the control. Minimum root length was observed in strain AH-45, which was 4.2% over the control. Statistical analysis confirmed that AH-34 showed a significant improvement in dry biomass of root over the control, which was 133.3%, followed by AH-46, AH-36, AH-49, and AH-44, which showed improvements by 114.3, 95.2, 81, and 81%, respectively, over the control. The lowest improvement was observed in dry biomass, which was 28.6%, where the AH-45 isolate was applied over the un-inoculated control.

**Table 3:** Impact of Fe-solubilizing isolates inoculants on maize growth, root colonization, and chlorophyll contents under axenic conditions.

Treatments	Root length	Shoot length	Shoot dry weight	Root dry weight	Chlorophyll a	Chlorophyll b	Carotenoids	Root colonization
	(cm)		(g jar^−1^)		(μg g^−1^ FW)			cfu × 10^5^ cm^−1^
Control	11.8 ± 0.058^i^	32.2 ± 0.635^l^	0.106 ± 0.03^g^	0.070 ± 0.0471^h^	0.57 ± 0.033^g^	0.57 ± 0.067^g^	0.97 ± 0.033^j^	18.3 ± 0.333^i^
AH-32	15.3 ± 0.033^de^	37.6 ± 0.1^fg^	0.137 ± 0.03^d-f^	0.123 ± 0.0262^c-e^	0.63 ± 0.058^fg^	0.64 ± 0.058^e^	1.05 ± 0.033^de^	33.0 ± 0.577^d^
AH-31	14.9 ± 0.384^e^	38.3 ± 0.058^f^	0.140 ± 0.06^c-f^	0.123 ± 0.027^c-e^	0.67 ± 0.033^c-f^	0.67 ± 0.058^cd^	1.04 ± 0.058^ef^	34.6 ± 0.333^cd^
AH-42	13.3 ± 0.033^g^	34.5 ± 0.088^ij^	0.123 ± 0.03^fg^	0.107 ± 0.0272^e-g^	0.61 ± 0.058f^g^	0.62 ± 0.033^ef^	1.0 ± 0.058^h-j^	23.3 ± 0.333^fg^
AH-38	12.8 ± 0.058^h^	33.4 ± 0.088^k^	0.120 ± 0.03^fg^	0.093 ± 0.0273^g^	0.60 ± 0.058^fg^	0.60 ± 0.033^fg^	0.99 ± 0.12^ij^	21.3 ± 0.333^gh^
AH-34	16.7 ± 0.033^a^	45.6 ± 0.088^a^	0.183 ± 0.03^a^	0.163 ± 0.0252^a^	0.78 ± 0.058^a^	0.79 ± 0.033^a^	1.13 ± 0.033^a^	49.3 ± 0.333^a^
AH-46	16.7 ± 0.058^a^	43.3 ± 0.058^b^	0.163 ± 0.03^ab^	0.150 ± 0.0267^ab^	0.76 ± 0.058^ab^	0.76 ± 0.067^a^	1.12 ± 0.033^ab^	48.3 ± 0.333^a^
AH-45	12.3 ± 0.058^hi^	34.2 ± 0.058^jk^	0.120 ± 0.06^fg^	0.090 ± 0.0256^g^	0.59 ± 0.058^fg^	0.60 ± 0.033^fg^	0.98 ± 0.058^j^	20.0 ± 0.577^hi^
AH-36	16.5 ± 0.033^ab^	42.4 ± 0.058^c^	0.163 ± 0.03^ab^	0.137 ± 0.0278^bc^	0.67 ± 0.058^c-f^	0.72 ± 0.088^b^	1.12 ± 0.058^ab^	48.0 ± 0.577^a^
AH-43	13.8 ± 0.033^fg^	35.1 ± 0.058^i^	0.127 ± 0.03^e-g^	0.100 ± 0.0241^fg^	0.62 ± 0.058^fg^	0.62 ± 0.033^ef^	1.00 ± 0.088^g-j^	25.0 ± 0.577^f^
AH-44	15.7 ± 0.033^bc^	40.3 ± 0.033^d^	0.160 ± 0.06^bc^	0.127 ± 0.0272^cd^	0.71 ± 0.058^a-d^	0.71 ± 0.088^b^	1.08 ± 0.058^cd^	41.6 ± 0.333^b^
AH-49	16.1 ± 0.033^cd^	40.3 ± 0.088^d^	0.153 ± 0.03^b-d^	0.127 ± 0.0272^cd^	0.72 ± 0.058^a-c^	0.72 ± 0.058^b^	1.09 ± 0.033^bc^	43.0 ± 0.577^b^
AH-51	15.7 ± 0.058^cd^	39.3 ± 0.033^e^	0.147 ± 0.03^b-e^	0.117 ± 0.0272^df^	0.70 ± 0.088^b-f^	0.70 ± 0.503^bc^	1.07 ± 0.033^cd^	37.0 ± 0.577^c^
AH-56	14.2 ± 0.058^f^	36.1 ± 0.058^h^	0.137 ± 0.03^d-f^	0.120 ± 0.0471^c-e^	0.62 ± 0.058^fg^	0.63 ± 0.088^ef^	1.02 ± 0.058^f-i^	25.0 ± 0.577^f^
AH-59	15.0 ± 0.058^e^	37.2 ± 0.033^g^	0.137 ± 0.03^d-f^	0.117 ± 0.0272^d-f^	0.64 ± 0.058^d-g^	0.65 ± 0.088^de^	1.03 ± 0.033^e-g^	34.0 ± 0.577^d^
AH-54	14.3 ± 0.033^f^	36.3 ± 0.033^h^	0.133 ± 0.03^d-f^	0.113 ± 0.0272^d-f^	0.63 ± 0.058^e-g^	0.64 ± 0.088^e^	1.03 ± 0.058^e-h^	28.3 ± 0.333^e^
LSD (*p* ≤ 0.05)	0.55	0.89	0.02	0.01	0.07	0.03	0.03	2.47

The findings listed in Table 4 confirmed that inoculation of Fe-solubilizing isolates improved the root colonization of maize under controlled conditions. These isolates showed significant improvement in root colonization over the un-inoculated control. The rhizobacterial isolate AH-34 shows maximum root colonization, which was 49 × 10^5^ cfu g^−1^ root, and a further increase was observed in isolates AH-46, AH-36, AH-49, and AH-44, which were shown to be 48 × 10^5^ cfu g^−1^ root, 48 × 10^5^ cfu g^−1^ root, 43 × 10^5^ cfu g^−1^ root, 42 × 10^5^ cfu g^−1^ root, respectively, over control. The lowest increase observed in all the tested treatments is AH-45, which was 20 × 10^5^ cfu g^−1^ root. However, all isolates used in this trial showed a significant improvement in root colonization over the un-inoculated control.

**Table 4:** Evaluating the qualitative Fe-solubilization index and efficiency of bacterial isolates.

Bacterial isolates (Fe)	HZD (mm)	CD (mm)	SE (%)	SI
Wheat				
AH-17	16 ± 0.29 ^cd^	10 ± 0.58 ^ab^	160 ± 6.4 ^a-c^	2.6 ± 0.06 ^a-c^
AH-19	15 ± 0.58 ^d^	10 ± 0.58 ^ab^	150 ± 14.6 ^bc^	2.5 ± 0.12 ^bc^
AH-22	19 ± 0.29 ^a^	10 ± 0.88 ^ab^	190 ± 14.3 ^a^	2.9 ± 0.12 ^a^
AH-26	18 ± 0.58 ^ab^	11 ± 0.58 ^a^	163 ± 11.8 ^ab^	2.63 ± 0.1 ^ab^
AH-29	13 ± 0.5 ^e^	10 ± 0.58 ^ab^	130 ± 12.5 ^c^	2 ± 0.1 ^c^
Maize				
AH-34	19 ± 0.29 ^a^	10 ± 0.33 ^ab^	190 ± 8.2 ^a^	2.9 ± 0.07 ^a^
AH-36	17 ± 0.58 ^bc^	10 ± 0.58 ^ab^	170 ± 9.1 ^ab^	2.71 ± 0.07 ^ab^
AH-44	13 ± 0.58 ^e^	8 ± 0.58 ^c^	162 ± 11.1 ^ab^	2.62 ± 0.09 ^ab^
AH-46	19 ± 0.76 ^a^	11 ± 0.58 ^a^	172 ± 5.7 ^ab^	2.72 ± 0.05 ^ab^
AH-49	15 ± 0.58 ^d^	9 ± 0.58 ^bc^	166 ± 4.3 ^ab^	2.66 ± 0.04 ^ab^
LSD (*p* ≤ 0.05)	1.759	1.547	30.64	0.3077

Values represent mean ± SE of 3 replications. *HZD, halo zone diameter, CD, colony diameter, SE, solubilizing efficiency, SI, solubilizing index.

### 3.6 Impact of Fe solubilizing isolates on physiological attributes of maize

Data shown in Table 4 confirmed that the application of Fe-solubilizing bacterial isolates increases chlorophyll a, chlorophyll b, and carotenoid in maize plants under a controlled environment. The highest improvement in chlorophyll a, chlorophyll b, and carotenoid was observed in the AH-34 isolate, which was shown to be 37.2, 37, and 16.4%, respectively, over the un-inoculated control. While the subsequent increase was observed in AH-46, AH-36, AH-49, and AH-44, which showed chlorophyll a content was 33.1, 23.3, 25.6, and 25%, chlorophyll b contents were 31.8, 24.9, 24.9, and 23.1% and carotenoid contents were 15.4, 14.7, 12.3, and 10.6%, respectively, improvement over control.

### 3.7 Fe-solubilization index and efficiency

The results were prominent in terms of Fe-solubilization, halo zone diameter, colony diameter, solubilizing efficiency, and solubilizing index. The isolates AH-22 and AH-26 isolated from wheat rhizosphere showed maximum halo zone diameter (19 and 18 mm), colony diameter (10 and 11 mm), solubilizing efficiency (190 and 163%), solubilizing index (2.9 and 2.63 mm) and AH-34, AH-36, and AH-46 from maize rhizosphere showed the highest Fe-solubilizing halo zone diameter (19, 17, and 19 mm), colony diameter (10, 10, and 11 mm), solubilizing efficiency (190, 170, and 172%), and solubilizing index (2.9, 2.71, and 2.72) (Table 4). The Fe-solubilizing assay revealed that isolates AH-22 and AH-26 from wheat, and AH-34, AH-36, and AH-46 from maize, showed excellent results of Fe-solubilization, which are statistically similar. The remaining isolates were not selected due to weak Fe-solubilization/growth-promoting attributes.

### 3.8 Characterization of Fe-solubilizing isolates

After the confirmation of the results under controlled conditions in the jar trial, five isolates—three from maize: AH-34, AH-36, and AH-46; two from wheat: AH-22 and AH-26— the best Fe solubilizing isolates perform the highest results, and further these isolates are examined *in vitro* for plant growth-promoting characteristics. The data presented in Table 5 are the *in vitro* characterization of the five best rhizobacterial isolates. All the examined bacterial isolates performed positive results regarding protease activity, hydrogen cyanide production (HCN), catalase activity, and oxidase activity. When H_2_O_2_ was drop by drop poured on the fresh bacterial colony, the formation of bubbles confirmed the positive catalase activity. The cellulose degradation ability was only positive in AH-22, AH-34, and AH-46 and was negative in AH-26 and AH-36, while the positive ACC-deaminase ability was observed in all tested isolates. Positive results were observed in all tested isolates regarding the indole-3-acetic acid (IAA) test with and without L-tryptophan. The AH-22 bacterial isolate showed the highest IAA production results, which is 22 μg mL-1 with L-tryptophan. AH-26, AH-34, AH-46, and AH-36 bacterial strains also showed prominent results in the presence of L-tryptophan at 20 μg mL^−1^, 18 μg mL^−1^, 18 μg mL^−1^, and 17 μg mL^−1^. In the absence of L-tryptophan, the highest results were observed in AH-22 and AH-46, which were found to be 16 μg mL^−1^. The rhizobacterial isolates AH-34 and AH-36 also show similar results, that is, 15 μg mL^−1^, and the AH-26 strain showed 13 μg mL^−1^ of IAA production without L-tryptophan.

**Table 5:** *In vitro* characterization of selected Fe solubilizing rhizobacterial isolates for increasing plant growth and biochemical traits.

Characters		AH-22	AH-26	AH-34	AH-46	AH-36
Protease activity		+	+	+	+	+
Hydrogen cyanide production (HCN)		+	+	+	+	+
Cellulose degradation activity		+	−	+	+	−
Oxidase activity		+	+	+	+	+
Catalase activity		+	+	+	+	+
ACC-deaminase activity		+	+	+	+	+
IAA production (μg/mL)	With L-tryptophan	22 ± 2.89^a^	20 ± 1.89^ab^	18 ± 2.65^a-c^	18 ± 1.53^a-c^	17 ± 1.15^b-d^
	Without L-tryptophan	16 ± 1.44^b-d^	13 ± 1.27^d^	15 ± 1.5^cd^	16 ± 1.1^b-d^	15 ± 0.98^cd^

Positive sign (+) showed the character availability, and negative sign (−) showed the absence of the character.

### 3.9 Identification of Iron-solubilizing isolates

**Figure 3:**
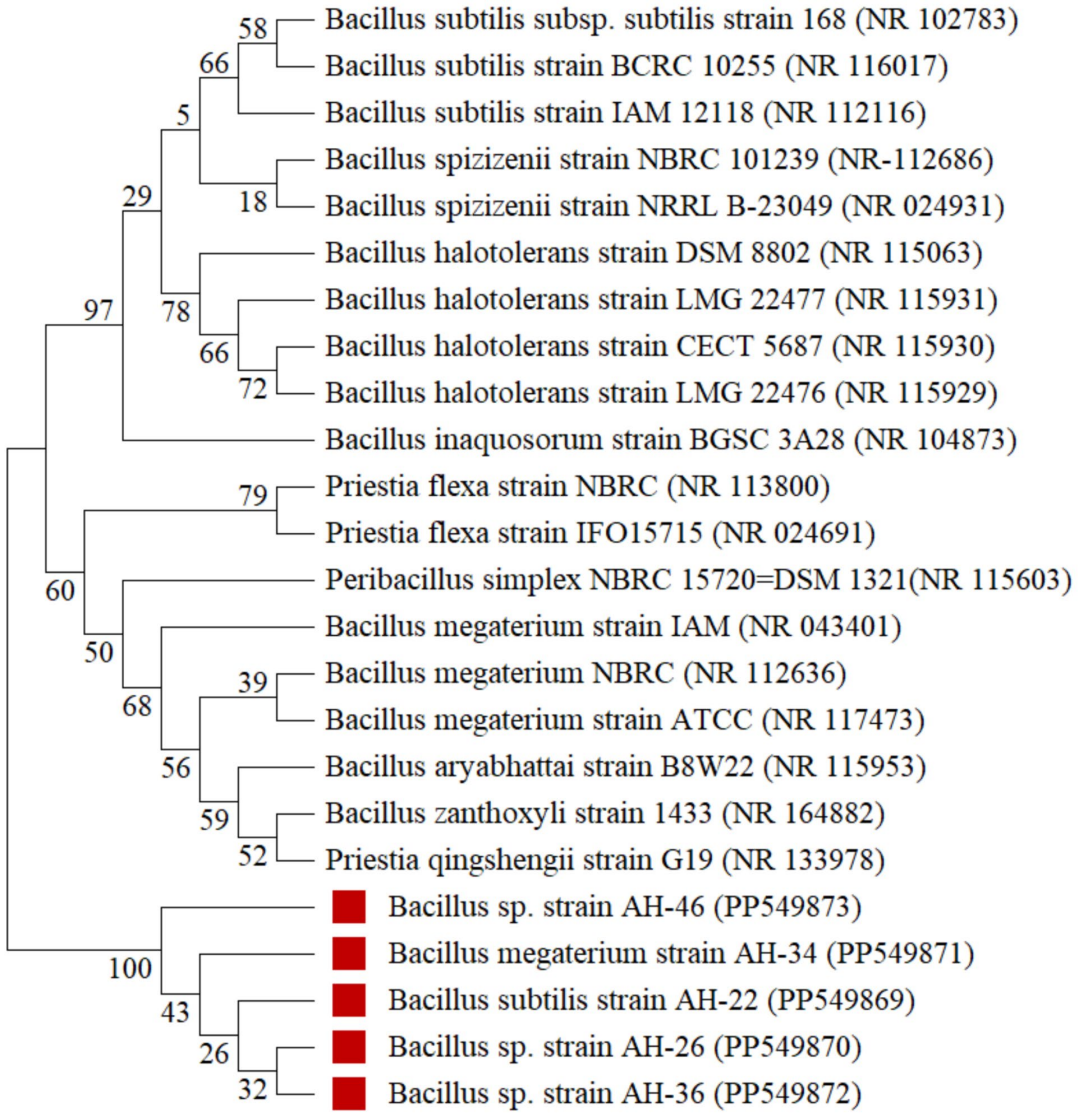
Neighbor joining tree of Fe solubilizing isolates *Bacillus subtilis* (AH-22), *Bacillus* sp. (AH-26), *Bacillus megaterium* (AH-34), *Bacillus* sp. (AH-36), *Bacillus* sp. (AH-46) (accession number: PP549869, PP549870, PP549871, PP549872, PP549873). The numbers displayed on the nodes (branch points) of a Neighbor-Joining (NJ) tree represent bootstrap values, a statistical measure of confidence or reliability in the branching pattern of the tree.

Identification of iron solubilizing isolates isolated from wheat and maize rhizosphere, AH26, AH34, AH36, and AH46, through 16S rRNA partial gene sequencing is presented in Figure 3. The rhizobacterial isolate AH-26 showed 99.85% resemblance to *Bacillus* sp. The bacterial isolate data were submitted further to NCBI with accession number PP549870 (684 bp). The rhizobacteria isolated AH-36 and AH-46, which were 99.71% similar to *Bacillus* sp. These isolates were identified as *Bacillus* sp. and submitted to NCBI with accession numbers PP549872 (689 bp) and PP549873 (700 bp). AH-34 and AH-22 were similar to *Bacillus magaterium* and *B. subtilis* with 99.71 and 99.28% resemblance, respectively, and were submitted to NCBI with accession numbers PP549871 (687 bp) and PP549869 (750 bp), respectively.

### 3.10 Multivariate analysis to check the relationship between observed attributes in response to applied treatments

**Figure 4:**
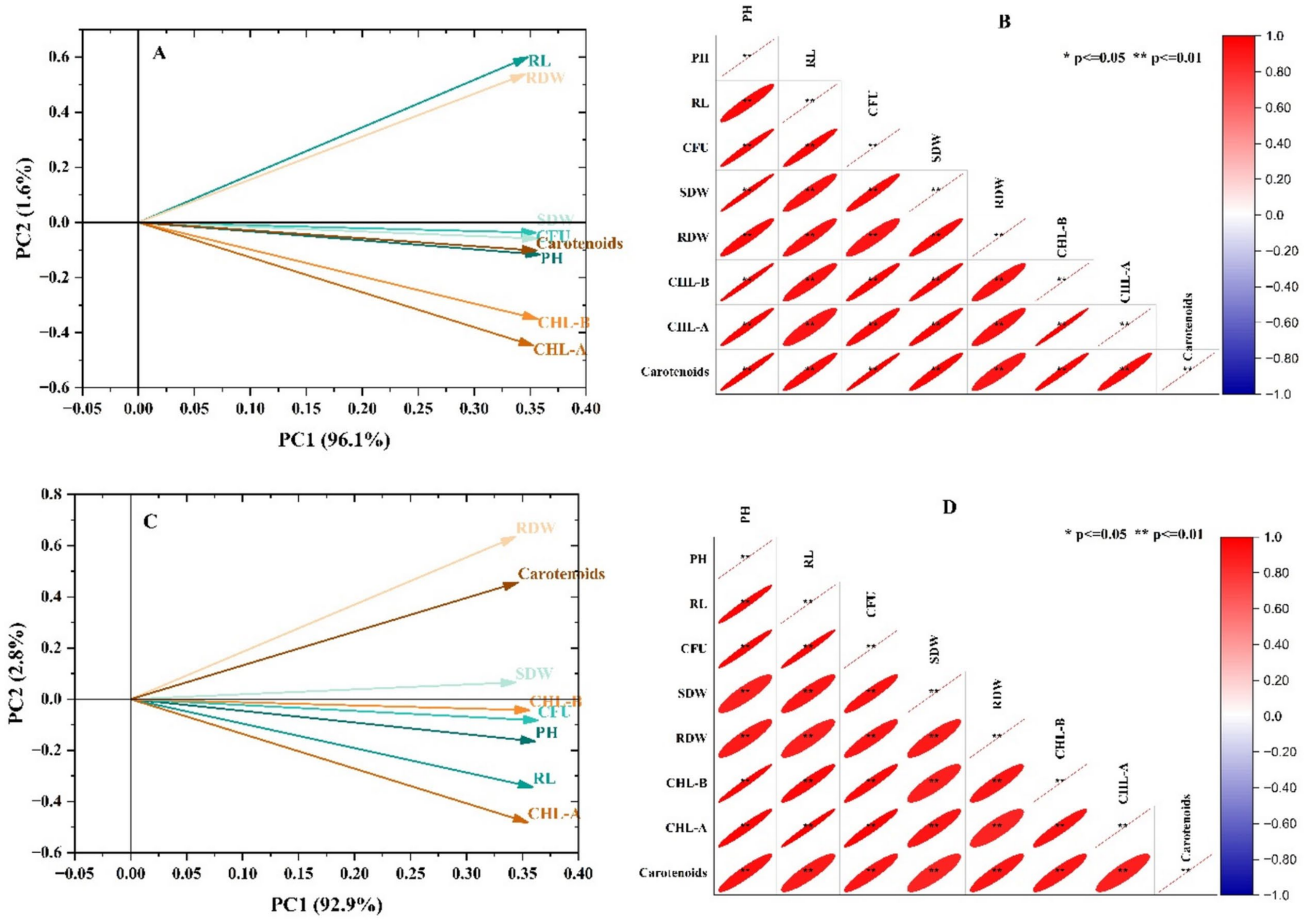
Multivariate analysis viz. **(A)** PCA of maize, **(B)** Pearson’s correlation of maize, **(C)** PCA of wheat, and **(D)** correlation of growth parameters of maize and wheat.

The relationship between the observed attributes of maize has been shown in Figure 4 in the form of Pearson correlation and PCA. The loading plot of PCA depicted that the first and second components showed 96.1 and 1.6% variation in growth and physiological attributes of maize (Figure 4A). Moreover, clustering of the inoculated set in the positive quadrant showed a positive impact of the application of Fe-solubilizing strains on maize growth. Moreover, Pearson’s correlation also described that the growth and physiological parameters were positively correlated, which depicted a significant positive impact of applied treatments on maize (Figure 4B). Similarly, the loading plot of PCA depicted that the first and second components showed 92.9 and 2.8% variations in growth and physiological attributes of wheat (Figure 4C). Moreover, clustering of the inoculated set in the positive quadrant showed a positive impact of the application of Fe-solubilizing strains on wheat growth. Pearson’s correlation also depicted a significant positive impact on the growth and physiological attributes of wheat (Figure 4D).

## 4 Discussion

Deficiency of Fe is a growing threat to humanity, health disturbance, physical/mental behavior, and other activities. Iron deficiency is a fast-spreading threat to the world. Plant growth-improving bacterial isolates secrete specific compounds that chelate Fe and have low molecular-weight compounds called siderophores reported by Khalid et al. (2015). These compounds can increase the availability and solubility of Fe to crop plants. Microbial-mediated biofortification through siderophore production is an emerging approach to reduce malnutrition by fortifying the most limited micronutrients Fe, Zn, and vitamin A in cereals (Kaur et al., 2020). He et al. (2020) stated that some microbes, which are linked with wheat primarily, are the rhizosphere microbes that synthesize siderophores and other important metabolites that enhance Fe solubility and plant uptake.

The current study was conducted to isolate, screen, and purify the siderophore-producing bacterial isolates from maize and wheat rhizosphere and then observed their impact on Fe-solubilization, plant growth-promoting traits, and improved growth of wheat and maize under axenic conditions. After character determination of microbes, it is observed that out of 30 isolates, each from wheat and maize, 15 isolates produced higher concentrations of siderophore, zinc solubilization, phosphorus solubilization, and exopolysaccharide production. Our findings are in line with that in the study by Kumari et al. (2022), which demonstrated that the SPS10 strain performed significantly and synthesized a competitively higher level of siderophore, which is 46.2 (SU%).

Under axenic conditions, wheat and maize growth trials showed an increase in root, shoot length, and dry weight where inoculation of seeds was performed over an un-inoculated control. Satish et al. (2020) confirmed that plant growth could be affected by the rhizobacterial inoculation in roots. Jar trial study depicted that 67.2 and 41.8% increase in shoot length, 34.6 and 41.8% in root length, 24.7 and 37.2% chlorophyll a, 30.1 and 37% chlorophyll b, 41.7 and 16.4% carotenoid contents in wheat and maize after inoculating with siderophore-producing bacterial isolates. Such improved growth attributes and physiological attributes provide a strong baseline to further check these Fe-solubilizing isolates on cereal crops in pot and field conditions. These findings align with the results of Yadav et al. (2021). Similar increases were observed in the potato rhizosphere, as demonstrated by Mushtaq et al. (2021b). Plant-microbe interaction is the primary factor in determining plant health, productivity, and the fertility of the soil. Kabiraj et al. (2020) demonstrate that inoculation of bacteria can significantly improve the agronomic parameters, which ultimately help to decrease environmental pollution and production cost. Previously, scientists have demonstrated that siderophores increase the uptake of Fe in the plant body, which ultimately increases the chlorophyll content, photosynthetic rate, and leaf area (Mushtaq, 2021). The current situation may suggest that an improvement in physiological characteristics might be due to an improvement in the solubilization of phosphorus, uptake/solubilization/translocation of Fe, phytohormone, and auxin production (Mushtaq et al., 2021b). Plant growth regulators secreted by PGPRs exhibited an increase in leaf area, chlorophyll content, and sugar content, and can decrease the lipid peroxidation and oxidative stress in plants (Khan et al., 2019). The results of the present study are also related to the results of Ekin (2019). Mushtaq (2021) stated that bacterial isolates increase nutrient contents, plant development, plant physiological attributes, growth, and yield through numerous (direct–indirect) approaches to the production of hormones, containing (gibberellins, cytokinin, and auxin). Similar results were also presented by Prasanna et al. (2015). Wheat biofortification through the inoculation of seeds by siderophore-producing bacterial isolates is a substitute method to achieve the desired micronutrient shortage in the routine diet of humans, especially in rural populations (Riaz et al., 2020).

Fe-solubilizing bacteria were further characterized for indole acetic acid production (IAA) in the presence and absence of L-tryptophan, and these findings are related to the study of Dar et al. (2022) and Mumtaz et al. (2017), which described that plant rhizosphere bacterial isolates increase the synthesis of auxin by providing L-tryptophan in the growth-media over without L-tryptophan. IAA synthesis by bacterial isolates in the rhizosphere of maize and wheat is responsible for efficiently proliferating roots, which ultimately increase water contents and nutrient status in plants under both stress and non-stress environments (Voronina et al., 2023). All the tested isolates have positive capability of catalase, protease activity, and cellulose degradation under the agar plate method. Iqbal et al. (2020) also demonstrated similar results, detecting increased protease and catalase activity in various endophytic and rhizobacterial strains. They also described the cellulose degradation capability of *Bacillus* sp. Such kinds of enzymes can increase the growth of crops under stressful environments, particularly biotic stress (Vaishnay et al., 2020). Production of hydrogen cyanide (HCN) by these bacterial isolates is related to the results of Mumtaz et al. (2017), who also defined the positive HCN-producing *Bacillus aryabhattai* strains ZM31 and S10, and Shoukry et al. (2018), who defined the oxidase, siderophore, and HCN production in maize and wheat rhizosphere. Moreover, plant growth-improving bacteria, along with ACC-deaminase production, are supportive for plants to decrease abiotic stress, induce raised levels of ethylene, and increase the growth of plants by ACC hydrolysis (ethylene precursor) into *α*-ketobutyarate and ammonia (Arshad et al., 2007).

The findings of this study can provide a sustainable method for improving agricultural productivity in natural conditions by enhancing nutrient bioavailability, their use efficiencies, reducing chemical inputs, improving soil health, and reducing environmental pollution under wheat and maize production. However, the use of Fe-solubilizing bacteria on a large scale might face certain challenges, namely, variability in colonization and survival of the bacteria under different environmental conditions and a lack of farmer’s awareness. However, these challenges can be addressed through formulation stability, field efficacy, regulatory oversight, and ecological safety through integrated research and policy support.

## 5 Conclusion

In conclusion, the selected rhizobacterial strains (AH-34, AH-36, and AH-46 for maize and AH-22 and AH-26 for wheat) effectively enhanced seedling growth, root colonization, and physiological traits of wheat and maize under axenic conditions. These strains show strong potential for use as cost-effective, eco-friendly biofertilizers to alleviate iron deficiency, promote sustainable crop production, and can be further studied for the development of carrier-based (compost or chemical fertilizer) formulation for sustainable production and biofortification of wheat and maize after large-scale testing in different agro-ecological zones.

## Data Availability

The datasets presented in this study can be found in online repositories. The names of the repository/repositories and accession number(s) can be found below: https://www.ncbi.nlm.nih.gov/genbank/, PP549869, PP549870, PP549871, PP549872, and PP549873.

## Footnotes

1
https://blast.ncbi.nlm.nih.gov/Blast.cgi

## References

[ref1] Ahmed E. Holmström S. J. (2014). Siderophores in environmental research: roles and applications. Microb. Biotechnol. 7, 196–208. doi: 10.1111/1751-7915.12117, PMID: 24576157 PMC3992016

[ref2] Ahsin M. Hussain S. Rengel Z. Amir M. (2020). Zinc status and its requirement by rural adults consuming wheat from control or zinc-treated fields. Environ. Geochem. Health 42, 1877–1892. doi: 10.1007/s10653-019-00463-8, PMID: 31696401

[ref3] Al-Adham I. Haddadin R. Collier P. (2013). “Types of microbicidal and microbistatic agents” in Russell, Hugo & Ayliffe's: Principles and practice of disinfection, preservation and sterilization. eds. Fraise A. P. Maillard J.-Y. Sattar S. A. (Hoboken, New Jersey, USA: Wiley-Blackwell), 5–70.

[ref4] Anwar Z. Basharat Z. Bilal Hafeez M. Zahra N. Rafique Z. Maqsood M. (2022). Biofortification of maize with zinc and iron not only enhances crop growth but also improves grain quality. Asian J. Agric. Biol. 2022:202102079 doi: 10.35495/ajab.2021.02.079

[ref5] Arnon D. I. (1949). Copper enzymes in isolated chloroplasts. Polyphenoloxidase in *Beta vulgaris*. Plant Physiol. 24, 1–15. doi: 10.1104/pp.24.1.1, PMID: 16654194 PMC437905

[ref6] Arshad M. Saleem M. Hussain S. (2007). Perspectives of bacterial ACC deaminase in phytoremediation. Trends Biotechnol. 25, 356–362. doi: 10.1016/j.tibtech.2007.05.005, PMID: 17573137

[ref7] Cappuccino J. G. Sherman N. (2002). Microbiology; a laboratory manual. 6th Edn. San Francisco, CA: Pearson education Inc.

[ref8] Chernin L. S. Winson M. K. Thompson J. M. Haran S. Bycroft B. W. Chet I. . (1998). Chitinolytic activity in *Chromobacterium violaceum*: substrate analysis and regulation by quorum sensing. J. Bacteriol. 180, 4435–4441. doi: 10.1128/jb.180.17.4435-4441.1998, PMID: 9721280 PMC107452

[ref9] da Silva Lopes K. Yamaji N. Rahman M. O. Suto M. Takemoto Y. Garcia-Casal M. N. . (2021). Nutrition-specific interventions for preventing and controlling anaemia throughout the life cycle: an overview of systematic reviews. Cochrane Database Syst. Rev. 9:CD013092. doi: 10.1002/14651858.CD013092.pub2, PMID: 34564844 PMC8464655

[ref10] Dar A. Were E. Hilger T. Zahir Z. A. Ahmad M. Hussain A. . (2022). Bacterial secondary metabolites: possible mechanism for weed suppression in wheat. Can. J. Microbiol. 69, 103–116. doi: 10.1139/cjm-2022-0181 36379032

[ref11] Dimkpa C. O. Merten D. Svatoš A. Büchel G. Kothe E. (2009). Siderophores mediate reduced and increased uptake of cadmium by *Streptomyces tendae* F4 and sunflower (*Helianthus annuus*), respectively. J. Appl. Microbiol. 107, 1687–1696. doi: 10.1111/j.1365-2672.2009.04355.x, PMID: 19457036

[ref12] Ehmann A. (1977). The Van Urk-Salkowski reagent a sensitive and specific chromogenic reagent for silica gel thin-layer chromatographic detection and identification of indole derivatives. J. Chromatogr. A 132, 267–276. doi: 10.1016/S0021-9673(00)89300-0, PMID: 188858

[ref13] Ekin Z. (2019). Integrated use of humic acid and plant growth promoting rhizobacteria to ensure higher potato productivity in sustainable agriculture. Sustainability 11:3417. doi: 10.3390/su11123417

[ref14] El-Sayed W. S. Akhkha A. El-Naggar M. Y. Elbadry M. (2014). In vitro antagonistic activity, plant growth promoting traits and phylogenetic affiliation of rhizobacteria associated with wild plants grown in arid soil. Front. Microbiol. 5:651. doi: 10.3389/fmicb.2014.00651, PMID: 25538687 PMC4255609

[ref15] Fasim F. Ahmed N. Parsons R. Gadd G. M. (2002). Solubilization of zinc salts by a bacterium isolated from the air environment of a tannery. FEMS Microbiol. Lett. 213, 1–6. doi: 10.1111/j.1574-6968.2002.tb11277.x, PMID: 12127480

[ref16] Felsenstein J. (1985). Confidence limits on phylogenies: an approach using the bootstrap. Evolution 39, 783–791. doi: 10.1111/j.1558-5646.1985.tb00420.x, PMID: 28561359

[ref17] Hassanisaadi M. Shahidi Bonjar G. H. Hosseinipour A. Abdolshahi R. Ait Barka E. Saadoun I. (2021). Biological control of *Pythium aphanidermatum*, the causal agent of tomato root rot by two *Streptomyces* root symbionts. Agronomy 11:846. doi: 10.3390/agronomy11050846

[ref18] He L. Yue Z.H. Chen C. Li C.Y. Li J. Sun Z.K. (2020). Enhancing iron uptake and alleviating iron toxicity in wheat by plant growth-promoting bacteria: theories and practices. *Intl J Agric Biol* 23, 190–196. doi: 10.17957/IJAB/15.1276

[ref19] Iqbal Z. Ahmad M. Jamil M. Akhtar M. F. U. Z. (2020). Appraising the potential of integrated use of *Bacillus* strains for improving wheat growth. Int. J. Agric. Biol. 24, 1439–1448. doi: 10.17957/IJAB/15.1581

[ref20] Kabiraj A. Majhi K. Halder U. Let M. Bandopadhyay R. (2020). “Role of plant growth-promoting Rhizobacteria (PGPR) for crop stress management” in Sustainable agriculture in the era of climate change. eds. Roychowdhury R. Choudhury S. Hasanuzzaman M. Srivastava S. (Cham: Springer), 367–389. doi: 10.1007/978-3-030-45669-6_17

[ref21] Kaur T. Rana K. L. Kour D. Sheikh I. Yadav N. Kumar V. . (2020). “Microbe-mediated biofortification for micronutrients: present status and future challenges” in New and future developments in microbial biotechnology and bioengineering. (Eds.) Rastegari, A. A., Yadav, A. N., and Yadav, N. (Elsevier: Amsterdam), 1–17. doi: 10.1016/B978-0-12-820528-0.00002-8

[ref22] Kaushal M. Sharma R. Vaidya D. Gupta A. Saini H. K. Anand A. . (2023). Maize: an underexploited golden cereal crop. Cereal Res. Commun. 51, 3–14. doi: 10.1007/s42976-022-00280-3

[ref23] Khalid S. Asghar H. N. Akhtar M. J. Aslam A. Zahir Z. A. (2015). Biofortification of iron in chickpea by plant growth promoting rhizobacteria. Pak. J. Bot. 47, 1191–1194.

[ref24] Khan N. Bano A. Rahman M. A. Guo J. Kang Z. Babar M. A. (2019). Comparative physiological and metabolic analysis reveals a complex mechanism involved in drought tolerance in chickpea (*Cicer arietinum* L.) induced by PGPR and PGRs. Sci. Rep. 9, 1–19. doi: 10.1038/s41598-019-38702-8 30765803 PMC6376124

[ref25] Kumar M. Prasanna R. Bidyarani N. Babu S. Mishra B. K. Kumar A. . (2013). Evaluating the plant growth promoting ability of thermotolerant bacteria and cyanobacteria and their interactions with seed spice crops. Sci. Hortic. 164, 94–101. doi: 10.1016/j.scienta.2013.09.014

[ref26] Kumar S. Stecher G. Suleski M. Sanderford M. Sharma S. Tamura K. (2024). MEGA12: molecular evolutionary genetic analysis version 12 for adaptive and green computing. Mol. Biol. Evol. 41:msae263. doi: 10.1093/molbev/msae263, PMID: 39708372 PMC11683415

[ref27] Kumari S. Kumar P. Kiran S. Kumari S. Singh A. (2022). Optimization of siderophore production by *Bacillus subtilis* DR2 and its effect on growth promotion of *Coriandrum sativum*. Russ. Agric. Sci. 48, 467–475. doi: 10.3103/S1068367422060076

[ref28] Lopez A. Cacoub P. Macdougall I. C. Peyrin-Biroulet L. (2016). Iron deficiency anaemia. Lancet 387, 907–916. doi: 10.1016/S0140-6736(15)60865-0, PMID: 26314490

[ref29] Lorck H. (1948). Production of hydrocyanic acid by bacteria. Plant Physiol. 1, 142–146. doi: 10.1111/j.1399-3054.1948.tb07118.x

[ref30] Mahuku G. S. (2004). A simple extraction method suitable for PCR-based analysis of plant, fungal, and bacterial DNA. Plant Mol. Biol. Report. 22, 71–81. doi: 10.1007/BF02773351

[ref31] Mehmood U. Inam-ul-Haq M. Saeed M. Altaf A. Azam F. Hayat S. (2018). A brief review on plant growth promoting rhizobacteria (PGPR): a key role in plant growth promotion. Plant Prot. 2, 77–82.

[ref32] Mumtaz M. Z. Ahmad M. Jamil M. Hussain T. (2017). Zinc solubilizing *Bacillus* spp. potential candidates for biofortification in maize. Microbiol. Res. 202, 51–60. doi: 10.1016/j.micres.2017.06.001, PMID: 28647123

[ref33] Mushtaq Z. (2021). PGPR: present role, mechanism of action and future prospects along bottlenecks in commercialization. Int. J. Environ. Qu. 41, 9–15. doi: 10.6092/issn.2281-4485/11103

[ref34] Mushtaq Z. Alasmari A. Demir C. Oral M. A. Bellitürk K. Baran M. F. (2024). Enhancing iron content in potatoes: a critical strategy for combating nutritional deficiencies. Potato Res. 2024, 1–27. doi: 10.1007/s11540-024-09758-5

[ref35] Mushtaq Z. Asghar H. N. Zahir Z. A. (2021a). Comparative growth analysis of okra (Abelmoschus esculentus) in the presence of PGPR and press mud in chromium contaminated soil. Chemosphere 262:127865. doi: 10.1016/j.chemosphere.2020.127865, PMID: 32791369

[ref9001] Mushtaq Z. Faizan S. Hussain A. (2021b). Role of Microorganisms as Biofertilizers. In Microbiota and biofertilizers. (Eds.) Hakeem, K. R., Dar, G. H., Mehmood, M. A., and Bhat, R. A. Cham: Springer. doi: 10.1007/978-3-030-48771-3_6

[ref36] Nathiya S. Janani R. Kannan V. R. (2020). Potential of plant growth promoting rhizobacteria to overcome the exposure of pesticide in *Trigonella foenum-graecum* (fenugreek leaves). Biocatal. Agric. Biotechnol. 23:101493. doi: 10.1016/j.bcab.2020.101493

[ref37] Oluwole O. Ibidapo O. Arowosola T. Raji F. Zandonadi R. P. Alasqah I. . (2023). Sustainable transformation agenda for enhanced global food and nutrition security: a narrative review. Front. Nutr. 10:e1226538. doi: 10.3389/fnut.2023.1226538 PMC10433737 37599683

[ref38] Penrose D. M. Glick B. R. (2003). Methods for isolating and characterizing ACC deaminase-containing plant growth-promoting rhizobacteria. Physiol. Plant. 118, 10–15. doi: 10.1034/j.1399-3054.2003.00086.x, PMID: 12702008

[ref39] Pikovskaya R. I. (1948). Mobilization of phosphorous in soil in connection with vital activity of some microbial species. Mikrobiologiia 17, 363–370.

[ref40] Prasanna R. Bidyarani N. Babu S. Hossain F. Shivay Y. S. Nain L. (2015). Cyanobacterial inoculation elicits plant defense response and enhanced Zn mobilization in maize hybrids. Cogent Food Agric. 1:998507. doi: 10.1080/23311932.2014.998507, PMID: 41180919

[ref41] Riaz U. Murtaza G. Anum W. Samreen T. Sarfraz M. Nazir M. Z. (2020). “Plant growth-promoting rhizobacteria (PGPR) as biofertilizers and biopesticides” in Microbiota and biofertilizers: A sustainable continuum for plant and soil health. eds. Hakeem K. R. Dar G. H. Mehmood M. A. Bhat R. A. (Cham: Springer International Publishing), 181–196.

[ref42] Sahay R. Patra D. D. (2014). Identification and performance of sodicity tolerant phosphate solubilizing bacterial isolates on *Ocimum basilicum* in sodic soil. Ecol. Eng. 71, 639–643. doi: 10.1016/j.ecoleng.2014.08.007

[ref43] Saitou N. Nei M. (1987). The neighbor-joining method: a new method for reconstructing phylogenetic trees. Mol. Biol. Evol. 4, 406–425. doi: 10.1093/oxfordjournals.molbev.a040454, PMID: 3447015

[ref44] Sarwar A. K. M. G. Biswas J. K. (2021). Cereal grains of Bangladesh–present status, constraints and prospects. Cereal Grains 1:19. doi: 10.5772/intechopen.97072

[ref45] Sarwar S. Khaliq A. Yousra M. Sultan T. (2022). Iron biofortification potential of siderophore producing rhizobacterial strains for improving growth, yield and iron contents of groundnut. J. Plant Nutr. 45, 2332–2347. doi: 10.1080/01904167.2022.2063733

[ref46] Satish L. Shamili S. Yolcu S. Lavanya G. Alavilli H. Swamy M. K. (2020). “Biosynthesis of secondary metabolites in plants as influenced by different factors” in Plant-derived bioactives: Production, properties and therapeutic applications . (Ed.) Swamy, M. (Springer Singapore), 61–100. doi: 10.1007/978-981-15-1761-7_3

[ref47] Schalk I. J. (2025). Bacterial siderophores: diversity, uptake pathways and applications. Nat. Rev. Microbiol. 23, 24–40. doi: 10.1038/s41579-024-01090-6, PMID: 39251840

[ref48] Schwyn B. Neilands J. (1987). Universal chemical assay for the detection and determination of siderophores. Anal. Biochem. 160, 47–56. doi: 10.1016/0003-2697(87)90612-9, PMID: 2952030

[ref49] Sheoran S. Kumar S. Ramtekey V. Kar P. Meena R. S. Jangir C. K. (2022). Current status and potential of biofortification to enhance crop nutritional quality: an overview. Sustainability 14:3301. doi: 10.3390/su14063301

[ref50] Shoukry A. A. El-Sebaay H. H. El-Ghomary A. E. (2018). Assessment of indole acetic acid production from *Rhizobium leguminosarum* strains. Curr. Sci. Int. 7, 60–69.

[ref51] Sierra G. A. (1957). A simple method for the detection of lipolytic activity of microorganisms and some observations on the influence of the contact between cells and fatty substrates. Antonie Van Leeuwenhoek 23, 15–22. doi: 10.1007/BF02545855, PMID: 13425509

[ref52] Simons M. Van Der Bij A. J. Brand I. De Weger L. A. Wijffelman C. A. Lugtenberg B. J. (1996). Gnotobiotic system for studying rhizosphere colonization by plant growth-promoting *Pseudomonas* bacteria. MPMI 9, 600–607. doi: 10.1094/mpmi-9-0600, PMID: 8810075

[ref53] Steel R. G. D. Torrie J. H. Dicky D. A. (1997). Principles and procedures of statistics- a biometrical approach. 3rd Edn. Singapore: McGraw-Hill Book International Co.

[ref54] Strieth D. Schwarz A. Stiefelmaier J. Erdmann N. Muffler K. Ulber R. (2021). New procedure for separation and analysis of the main components of cyanobacterial EPS. J. Biotechnol. 328, 78–86. doi: 10.1016/j.jbiotec.2021.01.007, PMID: 33484743

[ref55] Tamura K. Nei M. Kumar S. (2004). Prospects for inferring very large phylogenies by using the neighbor-joining method. Proc. Natl. Acad. Sci. USA 101, 11030–11035. doi: 10.1073/pnas.0404206101, PMID: 15258291 PMC491989

[ref56] Tanin M. J. Sharma A. Saini D. K. Singh S. Kashyap L. Srivastava P. . (2022). Ascertaining yield and grain protein content stability in wheat genotypes having the Gpc-B1 gene using univariate, multivariate, and correlation analysis. Front. Genet. 13:1001904. doi: 10.3389/fgene.2022.1001904, PMID: 36160017 PMC9490372

[ref57] Ul Hussan M. Saleem M. F. Hafeez M. B. Khan S. Hussain S. Ahmad N. . (2022). Impact of soil applied humic acid, zinc and boron supplementation on the growth, yield and zinc translocation in wheat. Asian J. Agric. Biol. 2022, 1–8. doi: 10.35495/ajab.2021.02.080

[ref58] Vaishnay A. Singh J. Singh P. Rajput R. S. Singh H. B. Sharma B. K. (2020). *Sphingobacterium* sp. BHU-AV3 induces salt tolerance in tomato by enhancing antioxidant activities and energy metabolism. Front. Microbiol. 11:443. doi: 10.3389/fmicb.2020.00443 32308647 PMC7145953

[ref59] Vasconcelos M. W. Gruissem W. Bhullar N. K. (2017). Iron biofortification in the 21^st^ century: setting realistic targets, overcoming obstacles, and new strategies for healthy nutrition. Curr. Opin. Biotechnol. 44, 8–15. doi: 10.1016/j.copbio.2016.10.001, PMID: 27780080

[ref60] Vazquez P. Holguin G. Puente M. E. Lopez-Cortes A. Bashan Y. (2000). Phosphate solubilizing microorganisms associated with the rhizosphere of mangroves growing in a semiarid coastal lagoon. Biol. Fertil. Soils 30, 460–468. doi: 10.1007/s003740050024

[ref61] Voronina E. Sokolova E. Tromenschleger I. Mishukova O. Hlistun I. Miroshnik M. . (2023). Properties of potential plant-growth-promoting bacteria and their effect on wheat growth promotion (*Triticum aestivum*) and soil characteristics. Microbiol. Res. 15, 20–32. doi: 10.3390/microbiolres15010002

[ref62] Wellburn A. R. (1994). The spectral determination of chlorophylls a and b, as well as total carotenoids, using various solvents with spectrophotometers of different resolution. J. Plant Physiol. 144, 307–313. doi: 10.1016/S0176-1617(11)81192-2

[ref63] Wollum A. G. (1982). “Cultural methods for soil microorganisms” in Methods of soil analysis: Part 2, chemical and microbiological properties, (Ed.) Page, A. L. (John Wiley & Sons, Hoboken), 9, 781–802. doi: 10.2134/agronmonogr9.2.2ed.c37

[ref64] Xie H. Zhang Y. Wu Z. Lv T. (2020). A bibliometric analysis on land degradation: current status, development, and future directions. Land 9:28. doi: 10.3390/land9010028

[ref65] Yadav R. Ror P. Rathore P. Kumar S. Ramakrishna W. (2021). Bacillus subtilis CP4, isolated from native soil in combination with arbuscular mycorrhizal fungi promotes biofortification, yield and metabolite production in wheat under field conditions. J. Appl. Microbiol. 131, 339–359. doi: 10.1111/jam.14951, PMID: 33269514

[ref66] Zhang Y. Y. Stockmann R. Ng K. Ajlouni S. (2022). Revisiting phytate-element interactions: implications for iron, zinc and calcium bioavailability, with emphasis on legumes. Crit. Rev. Food Sci. Nutr. 62, 1696–1712. doi: 10.1080/10408398.2020.1846014, PMID: 33190514

[ref67] Zulfiqar U. Maqsood M. Hussain S. Anwar-ul-Haq M. (2020). Iron nutrition improves productivity, profitability, and biofortification of bread wheat under conventional and conservation tillage systems. J. Soil Sci. Plant Nutr. 20, 1298–1310. doi: 10.1007/s42729-020-00213-1

